# CDK 4/6 inhibitor induced lung injury: a case report and review of literature

**DOI:** 10.3332/ecancer.2021.1245

**Published:** 2021-06-07

**Authors:** Namrata Mathew, Anjana Joel, Anand George Andrews, Ajoy Oommen John, Ashish Singh

**Affiliations:** Department of Medical Oncology, Christian Medical College and Hospital, Ida Scudder Road, Vellore 632004, Tamilnadu, India

**Keywords:** drug induced pneumonitis, CDK 4/6 inhibitors, palbociclib

## Abstract

Palbociclib is a cyclin dependent kinase (CDK) 4/6 inhibitor that is indicated in combination with an aromatase inhibitor for first-line treatment of hormone receptor-positive, human epidermal growth factor receptor 2 -negative advanced or metastatic breast cancer. The commonly described side effects of palbociclib are neutropenia, anaemia, thrombocytopenia, fatigue, nausea, stomatitis, alopecia, diarrhoea, decreased appetite, vomiting, asthenia, peripheral neuropathy and epistaxis. However, post approval, increasing use of this drug has revealed another potentially fatal complication, in the form of pneumonitis, especially in the Asian population. The PALOMA 3 trial showed that rates of grade 3 and grade 4 adverse events were modestly higher in Asians than non-Asians, though palbociclib exposure was similar in both races. From this, we could infer that adverse effects of this drug must be monitored more specifically in individual racial populations. We report a patient who developed pneumonitis while on palbociclib and discuss the possible mechanisms and management of CDK 4/6 inhibitor-related lung injury.

## Introduction

Cyclin dependent kinase (CDK) 4/6 inhibitors are the backbone of the treatment of metastatic oestrogen receptor positive, human epidermal growth factor receptor 2 (HER2)-negative breast cancer. The haematological adverse events (neutropenia, leukopenia, thrombocytopenia) and non-haematological adverse events (fatigue, diarrhoea, nausea) are well described. We describe a patient who developed pneumonitis while on palbociclib, review the literature and discuss the possible mechanisms and management of CDK 4/6 inhibitor related lung injury.

## Case presentation

**Figure 1.**
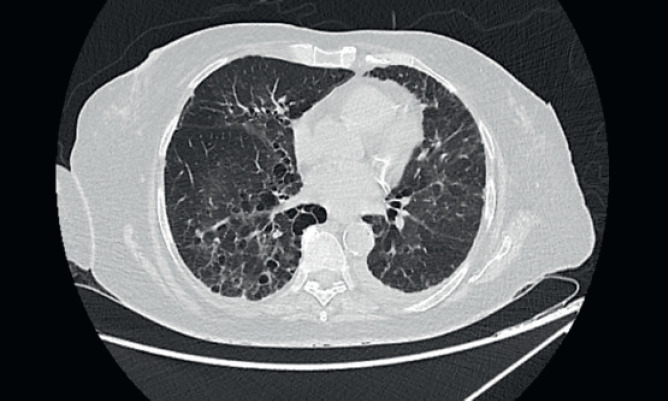
High-resolution CT thorax of the patient prior to initiation of CDK 4/6 inhibitor (Palbociclib).

A 67-year-old post-menopausal lady was diagnosed to have metastatic hormone positive breast cancer in February 2019. Her imaging showed multiple bony metastases with few bilateral subpleural metastases (<1 cm). She underwent open reduction and internal fixation for a pathological fracture of her right neck of femur. Biopsy of the breast lump showed an infiltrating ductal carcinoma grade 1, Estrogen receptor (ER) + (Allred 8/8), progesterone receptor+ (PR+) and HER2 negative. She was a well-controlled hypertensive and had a background of bronchiectasis (Figure 1) for which she was asymptomatic. Her bronchiectasis was first diagnosed in 2 years prior to the current diagnosis of breast cancer, when she presented to another hospital was a probable infective exacerbation of underlying lung disease. She had no prior history of necrotising pneumonia in childhood or tuberculosis or features of ciliary dyskinesias/cystic fibrosis. There was no clinical or serological evidence of autoimmune disease (acute inflammatory markers were, however, elevated due to inflammatory, fumigating breast carcinoma). Acute infective aetiologies were also ruled out, with multiple blood and sputum cultures for bacteria, mycobacteria and fungi yielding no growth. Polymerase chain reaction (PCR) for viruses and mycobacteria was also negative. There was no evidence of immunocompromise other than underlying malignancy which was diagnosed subsequently in Feb 2019.

**Figure 2.**
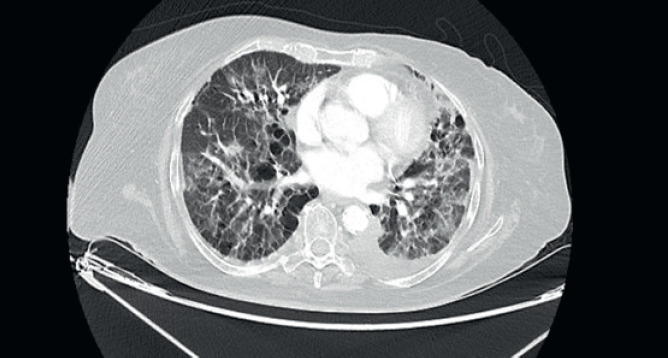
High-resolution CT thorax of the patient, taken at timepoint of clinical deterioration, three months after initiation of CDK 4/6 inhibitor (Palbociclib) therapy.

She was started on 3-monthly Zoledronic acid and palbociclib with Letrozole. There was significant reduction in size of the breast lesion, with healing of the breast ulcer and clinical improvement in terms of her bone pains. Four months later, she developed dry nonproductive cough and worsening breathlessness on exertion with no history of fever or prodromal illness. She was non-neutropenic with no renal or hepatic dysfunction. Arterial blood gas showed hypoxaemia and type 1 respiratory failure. Her high-resolution computed tomography (CT) thorax (Figure 2) was compared with her baseline imaging (Figure 1) and showed multiple new patchy and confluent ground glass opacities (GGOs) in bilateral lung fields, suggestive of acute interstitial lung disease (ILD).

A comprehensive workup including blood and sputum cultures was negative for bacterial, mycobacterial, fungal infections, influenza and *pneumocystis carinii*. Serological workup for underlying connective tissue disorder including anti-nuclear antibody, double-stranded DNA, Rheumatoid factor and anti-cyclic citrullinated peptide (CCP) was also negative. She was started on noninvasive ventilation and broad-spectrum intravenous (IV) antibiotics as she was unfit for a bronchoscopy/bronchoalveolar lavage (BAL) in view of her rapid deterioration. After a detailed discussion with the patient, she was started on IV methylprednisolone with an empirical diagnosis of drug induced pneumonitis. She refused aggressive treatment including ICU care, invasive ventilation and continued non-invasive ventilation. She was weaned off the noninvasive ventilation and was discharged on home oxygen therapy and letrozole. A follow-up CT was not performed as the patient clinically improved on systemic corticosteroids and antibiotics and she refused subsequent scans and opted for comfort care. Thus, after initiation of palbociclib in March 2019, palbociclib was discontinued in June 2019; after she developed the pneumonitis. She had also expressed her wishes against further imaging or invasive procedures. Therefore, bronchoscopy, trans-bronchial lung biopsy and/or BAL were not performed. She succumbed to her illness a month later at home.

## Discussion

Drug induced pneumonitis related to conventional chemotherapeutic agents like bleomycin, methotrexate, gemcitabine and taxanes and oral agents like epidermal growth factor receptor tyrosine kinase inhibitors and everolimus is well described [1, 2]. Pneumonitis as an immune related adverse event, especially following anti-cytotoxic T lymphocyte associated protein (CTLA) 4 agents is managed with the help of an algorithmic approach [3]. Improvement in our understanding of the mechanisms and patterns of pulmonary toxicity related to these agents has reflected in our capacity to suspect and recognise this toxicity early. Delayed recognition of this entity leads to a more severe grade of pneumonitis at diagnosis which leads to higher morbidity and mortality.

CDK 4/6 inhibitors are known for their efficacy and favourable adverse event profile which allows patients to maintain their quality of life. Severe and adverse non-haematological toxicities associated with these drugs are rare and sporadic and have been only been described in case reports or case series. [4–6].

The incidence of pulmonary toxicity (pneumonitis/respiratory failure/embolism) ranges around 1% [7–14]. There are few prior publications reporting CDK 4/6 inhibitor associated ILD (Table 1). It is a diagnosis of exclusion, in the setting of patients on CDK 4/6 inhibitors presenting with respiratory distress without any infective or auto immune aetiology. All patients were managed with steroids, ventilatory support and supportive measures. The first report of CDK 4/6i related pneumonitis in Asia was from Japan among 14 patients, with the use of Abemaciclib [15].

Mechanisms for CDK 4/6 inhibitor related lung injury are largely unclear and are still under investigation. In a study done on mouse models in 2020, palbociclib (CDK 4/6 inhibitor) was evaluated as a means to ameliorate pulmonary fibrosis in mice treated with intra-tracheal bleomycin. It was postulated that as CDK 4/6 inhibitors were essentially cell cycle inhibitors, they could attenuate the development of bleomycin induced pulmonary fibrosis. However, though palbociclib in this study was found to reduce collagen deposition in the lung, it did not translate into improvement in pulmonary function and also led to significant weight loss and deterioration of general health in the treated mice. BAL showed recruitment of large number of inflammatory cells, including significantly increased levels of monocyte-derived and interstitial macrophages, dendritic cells, neutrophils, γδTCR+ and CD8+ effector T-cells.

It was further proposed that this altered inflammatory cell milieu, in the presence of a cell cycle inhibitor like palbociclib, led to cell senescence and a consequent ‘senescence associated secretory phenotype’ (SASP). The SASP is in essence an increase in inflammatory cytokines, growth factors and extracellular matrix modulating proteins that promote pulmonary fibrosis [2]. The role of cellular SASP has been elucidated in other studies as well [3, 4]. However, the possible synergy of palbociclib and Bleomycin, and the exact role of each of the above-mentioned inflammatory cell types in CDK 4/6 inhibitor mediated lung injury has to be addressed in future studies.

Our patient and those described in prior literature have developed these side effect months into their treatment, implying that this is not an immediate side effect following initiation of CDK 4/6i. There are no patient or disease related factors that point to a predisposition for CDK 4/6i related pneumonitis. The diagnosis of CDK 4/5 inhibitor related ILD in majority of these patients was arrived at after extensive workup (including a bronchoscopy with BAL fluid cultures and transbronchial lung biopsy) to rule out infections, autoimmune aetiologies and disease progression. As with any other drug induced pneumonitis, removal of the offending agent along with oxygen and ventilatory support with IV steroids and broad-spectrum antibiotic cover, while allowing recovery of pulmonary function is the common therapeutic approach.

## Conclusion

The use of CDK 4/6 inhibitors in the first- and second-line setting in metastatic hormone positive breast cancer is the new standard of care and has led to the more widespread use of these agents. CDK 4/6i related pneumonitis requires early diagnosis and prompt discontinuation of the offending agent in order to improve outcomes.

## Conflicts of interest

The authors declare that they have no conflicts of interest.

## Funding statement

The authors received no financial support for the research, authorship and publication of this article.

## Figures and Tables

**Table 1.** Comparison of our patient with those described *a priori*.

		CDK 4/6 inhibitor	Age	CT appearance	Companion drug	Bronchoscopy/transbronchial lung biopsy	Outcome
Patient 1	Jazieh *et al* [4]	Palbociclib	74	Bilateral ground glassing	Fulvestrant	Nonspecific lung injury	Recovered after 8 months of home oxygen
Patient 2	Jazieh *et al* [4]	Abemaciclib	60	Patchy multifocal alveolar ground-glass densities within the upperlobes	Fulvestrant	BAL showed inflammatory cells	Dead
Patient 3	Ahsan *et al* [5]	Palbociclib	52	Bilateral ground glassing	NA	Not done	Hospice
Patient 4	Gong *et al* [6]	Palbociclib	72	Bilateral ground glassing	Fulvestrant	Not done	Resolution after 3 months of home oxygen
Patient 5	Ofer *et al* [16]	Palbociclib	71	Peripheral sub-pleural consolidations with air bronchogram and scattered GGO	Letrozole	Transbronchial lung biopsy showed subacute lung injury/organising diffuse alveolar damage	Dead
Patient 6	Our patient	Palbociclib	67	Bilateral ground glassing	Letrozole	Not done	Dead
